# Increased Prevalence of Diabetes in Argentina Is Due to Easier Health Care Access Rather than to an Actual Increase in Prevalence

**DOI:** 10.1371/journal.pone.0092245

**Published:** 2014-04-03

**Authors:** Adolfo Rubinstein, Laura Gutierrez, Andrea Beratarrechea, Vilma E. Irazola

**Affiliations:** Center of Excellence, South American Center for Cardiovascular Health (SACECH), Institute for Clinical Effectiveness and Health Policy (IECS), Buenos Aires, Argentina; Gentofte University Hospital, Denmark

## Abstract

**Introduction:**

According to the Argentine National Risk Factor Survey (ANRFS), between 2005 and 2009, self-reported Diabetes increased in Argentina from 8.4% to 9.6%, accompanied by a raise in the prevalence of obesity and low physical activity. In the same period, it also increased blood sugar checks from 69.3% to 75.7%. Since surveillance data in Argentina rely on self-reports, the estimated prevalence of diabetes may be affected by an increase in the proportion of subjects with access to preventive services. We evaluated the independent effect of a recent blood sugar check, on the increase in self-reported diagnoses of diabetes between 2005 and 2009.

**Materials and Methods:**

A secondary analysis of data from the 2005 and 2009 ANRFS was performed. Diabetes was defined as having been diagnosed Diabetes or high blood sugar by a health professional, obesity was calculated as BMI≥30 kg/m^2^, based on self-reported height and weight and physical activity was measured using the International Physical Activity Questionnaire. We used logistic regression models to explore the relationship between prevalence of self-reported diabetes and recent blood sugar check as the main predictor.

**Results:**

The prevalence of diabetes rose from 8.4% to 9.6%; obesity from 14.5% to 18% and low physical activity from 46.2% to 55%, between 2005 and 2009. Among those who recently checked their blood sugar no differences were found in the prevalence of diabetes: 13% in 2005 vs. 13.2% in 2009. Findings of the multivariable analysis showed that obesity and low physical activity were significantly associated with self reported diabetes in the adjusted model (OR = 1.80 for obesity, and OR = 1.12 for low physical activity but the strongest predictor was recent blood sugar check (OR = 4.75).

**Discussion:**

An increased prevalence of self-reported diabetes between 2005 and 2009 might indicate an improvement in the access to preventive services rather than a positive increase in the prevalence of diabetes.

## Introduction

Diabetes mellitus (DM) is an important cause of mortality and morbidity worldwide, through both direct metabolic complications and increased mortality from cardiovascular and kidney diseases [1]. A recent systematic review of published articles and unpublished data sources identified through the WHO Global InfoBase showed that diabetes is a rising global hazard, with the number of adults increasing more than double over the last three decades. Although population growth and ageing seem to be the main contributors to this rise, there is also an important epidemiological component related to an age-adjusted global mean fasting plasma glucose (FPG) increase of at least by 0.07 mmol/L per decade [2]. In developing countries, the rising prevalence of diabetes has been linked to the obesity epidemic, with excess weight accounting for about 90% of Type 2 diabetes [3]. In Argentina, the prevalence of DM increased from 8.4% to 9.6% between 2005 and 2009, as reported by the two national population-based surveys of risk factors that took place over this period of time. This finding was accompanied by an increase in the prevalence of self-reported obesity and low physical activity in the same period [4].

However, the prevalence of DM was obtained by self-report during in-person interviews (physical or biochemical measures were not performed in both surveys). Self-reported diabetes prevalence may be biased because respondents may not be aware of their risk status. In effect, to be aware of any diagnoses, a subject needs not only an effective contact with a health care provider but also a good understanding and comprehension of her/his condition, particularly in the case of diseases like type 2 DM that might go unnoticed until a complication eventually arises along its course. Hence, these two aspects might affect diabetes awareness both as a consequence of barriers of access to health services and low health literacy skills, especially in individuals with low levels of education [5]. If low access is an issue, then the prevalence of DM could be underestimated, as reported both in the US, where one-half of diabetes cases were undiagnosed in uninsured Hispanic men and in a recent study from India, where prevalence of diabetes was grossly underestimated by self-reports [6]–[7].

On the other hand, since these surveys are based on the WHO STEPS approach, which focuses on obtaining core data with a standard methodology to determine major risk factors for surveillance purposes [8], this reported increase in the prevalence of diabetes, regardless of the actual prevalence if direct measures were obtained, should also reflect an increase in the disease burden attributable to this condition, and thus help inform policies to combat non-communicable diseases (NCD). Nonetheless, the same survey reported an increase from 69.3% in 2005 to 75.7% in 2009, in the frequency of individuals who reported at least one blood sugar check in the past [4]. This fact could indicate an improvement in access to preventative health services and hence more opportunities for those who have unknown diabetes to be diagnosed rather than an actual increase in the prevalence of this condition.

Therefore, the aims of this study were 1) to evaluate the independent effect of a recent blood sugar check, as reported by survey respondents, on the increase in self-reported diagnoses of diabetes between 2005 and 2009, and 2) to evaluate the effects of other known risk factors such as obesity and low physical activity.

## Materials and Methods

### Design and Population

Both surveys were obtained through anonymous forms that do not contain identifiable or potentially identifiable information. Additionally, this study does not involve merging these databases in such a way that individuals might be identified. According to national regulations, the data obtained from these surveys are public sources with unrestricted access and do not require informed consent from participants. The data sets have been made public by the Ministry of Health for research purposes and therefore, IRB review was not required for this study”.

We performed a secondary analysis of data obtained from the first wave (2005) and second wave (2009) of the Argentine National Risk Factor Survey (ANRFS). These cross-sectional surveys are repeated over time as part of the national surveillance system for NCDs. The ANRFS is a nationally representative survey that included 41,392 participants in 2005 and 34,732 participants in 2009 from all districts of the country, sampled through a probabilistic multi-stage process. The surveys were based on a complex sample design and system of weighting to compute population-based estimates of health conditions and behaviors. The response rate was 86.7% in 2005, and 79.8% in 2009. Risk factors and behavioral determinants as well as socioeconomic factors were self-reported by participants during in-person interviews. Methodological characteristics of the ANRFS have been published elsewhere [9], [10].

### Definition of the main variables

DM was defined as having been told by a health professional that the subject had diabetes or high blood sugar. Recent blood sugar check was defined as having at least one measure within 2 years of the survey (response options were: less than one year, one to two years, more than 2 years, and never). Obesity was calculated as a body mass index of 30 kg/m2 or higher, based on self-reported height and weight. Physical activity (PA) was measured using the International Physical Activity Questionnaire (IPAQ), a widely used and validated instrument for measuring levels of physical activity in healthy adult populations [11]. We compared respondents with low PA versus those with moderate and high PA.

Other demographic and socio-economic (SES) variables used in the models were: age (less than or older than 50 years old), sex, educational attainment (secondary school incomplete or completed and university education; completed or incomplete primary school was the reference category), and health insurance coverage status (Private or social health insurance versus public coverage only).

### Data Analysis

First, the prevalence of self-reported DM was calculated for 2005 and 2009 and then stratified by recent blood sugar check. Second, all subjects included in the 2005 (n = 41,392) and 2009 (n = 34,732) surveys were pooled (n = 76,124) and explanatory logistic regression models were developed, whereby different sets of variables were regressed on self-reported prevalence of DM. A four-step model was built: in the first model, year of the survey was included as a sole predictor. This was followed by a second model, where recent blood sugar check, the main predictor, was added. The third model contained the previous variables and added demographic and SES variables to adjust for potential confounders. The final model included all variables from the previous models, plus obesity and low PA. Interaction between recent blood sugar check and education was tested to assess the statistical significance of changes in the frequency of recent blood sugar checks by levels of education, between 2005 and 2009. Only cases with non-missing data for all variables were included in the models, leaving a final sample size of 68,245 subjects. All analyses were performed with PROC SURVEYFREQ and PROC SURVEYLOGISTIC in SAS 9.3 (SAS Institute Inc., Cary, NC, USA).

## Results

Descriptive characteristics of the main variables in the two surveys can be seen in Table 1. There were no important differences in age groups, sex and SES variables except for a slight increase in the proportion of subjects with a higher level of education in 2005 and with health insurance coverage in 2009.

**Table 1:** Characteristics of the participants enrolled in both surveys.

	ANRFS 2005‡	ANRFS 2009‡	P
	N = 41,392	N = 34,732	
Sex, n (% of males)	17,827 (43.1)	15,028 (43.3)	0.5794
Age, n (% of >50 years old)	14,662 (35.4)	12,817 (36.9)	<0.0001
Education			
Primary school completed, n (%)	15,548 (35.8)	12,430 (36.9)	<0.0001
Secondary school completed, n (%)	15,002 (36.2)	13,374 (38.8)	
Tertiary or University education, n (%)	10,842 (28)	8,928 (24.3)	
Health Insurance coverage, n (%)	27,194 (66.5)	24,431 (74.4)	<0.0001

‡ ANRFS: Argentine National Risk Factor Surveys at years 2005 and 2009.

As shown in Table 2, the prevalence of diabetes rose 14.1%, from 8.4% to 9.6%; obesity rose 23.3%, from 14.5% to 18.0% and low physical activity rose 19%, from 46.2% to 55%, between 2005 and 2009. However, the percentage of subjects who had a blood sugar check in the last 2 years since the survey also rose 15.6% between 2005 and 2009: from 56.4% to 65.2%. When we estimated the percentage of individuals who reported a diagnosis of diabetes only among those who had recently checked their blood sugar, we found no significant difference in the prevalence between 2005 and 2009: 13.0% in 2005 vs. 13.2% in 2009.

**Table 2:** Prevalence of Risk factors and main predictor variables.

Risk Factors (self-reported weighted prevalences)	Year 2005	Year 2009	P value
Diabetes, % [95% CI]	8.4 [7.8;9.1]	9.6 [9.1;10.1]	0.0027
Obesity, % [95% CI]	14.6 [13.9;15.4]	18.0 [17.3;18.7]	<0.0001
Low Physical activity, % [95% CI]	46.2 [45.1;47.3]	55.0 [54.2;55.9]	<0.0001
Recent blood sugar check, % [95% CI]‡	56.4 [55.2;57.5]	65.2 [64.4;66.0]	<0.0001

‡ Recent blood sugar check was defined as having at least one measure within 2 years of the survey.

The findings of the multivariable analysis are shown in Table 3. The dependent variable of the logistic regression models was self-reported diabetes. In model 1, we found an independent association of year of the survey (2009 vs. 2005) on the prevalence of diabetes (OR 1.16). In model 2, recent blood sugar check had an OR of 5.70, and year of the survey (2009 vs. 2005) was no longer retained as a significant factor. The OR of recent blood sugar check did not change after the inclusion of demographic and SES variables in model 3. Age >50 year old (OR = 2.54) and university education (OR = 0.49) were independently associated with self-reported diabetes. In model 4, the known predictors of diabetes, obesity and low physical activity, were incorporated. Both risk factors were significantly associated with the dependent variable in the multivariable adjusted model (OR = 1.80 for obesity, and OR = 1.12 for low physical activity). In this final model, despite being partially confounded by obesity, recent blood sugar check was still the strongest predictor of diabetes when compared to the other explanatory variables, with an OR = 4.75, as compared to 5.70 in earlier models (less than 20% attenuation of its effect).

**Table 3:** Four-step logistic regression models predicting self-reported diabetes.

Model I	Variable	OR [95% CI]	P
	Year (2009 vs. 2005)	1.16 [1.05;1.27]	0.0032

Model II	Variable	OR[95% CI]	P
Year of survey+main predictor	Year (2009 vs. 2005)	1.041 [0.94–1.15]	0.4429
	Recent Glycemic Control	5.70 [4.90–6.64]	<.0001

Model III	Variable	OR[95% CI]	P
Year of survey+main predictor+demographic and SES variables ‡	Year (2009 vs. 2005)	1.041 [0.98;1.20]	0.1203
	Recent blood sugar check	5.70 [(4.14;5.729]	<.0001
	Male gender	0.94 [0.847;1.040]	0.2249
	Older than 50 years old)	2.54 [2.27;2.84]	<.0001
	Secondary school	0.75 [0.67;0.84]	0.2090
	University education	0.49 (0.43; 0.56	<.0001
	Health insurance	0.89 (0.776–1.01)	0.0808

Model IV	Variable	OR[95% CI]	P
Year of survey+main predictor+demographic and SES variables+known risk factors	Year (2009 vs. 2005)	1.05 [0.95;1.16]	0.3681
	**Recent Glycemic Control**	**4.74 [4.03;5.56]**	**<.0001**
	Sex (males vs. females)	0.95 [0.85;1.05]	0.3104
	Age (older vs. younger than 50 years old)	2.41 [2.16–2.70]	<.0001
	Secondary vs. primary school or less	0.767 (0.68;0.86]	0.2501
	University education vs. primary school or less	0.52 [0.45;0.6,0]	<.0001
	Health insurance vs. no insurance	0.90 [0.79;−1.03]	0.1162
	**Low PA** ‡‡	**1.12 [1.01;1.24]**	**0.0378**
	**Obesity**	**1.80 [1.61;2.02]**	b

‡ demographic and SES variables: sex, age (% of >50 years old); education (% of primary school, secondary school, and terciary school or university); and health insurance coverage;

‡‡ Low PA: low Physical activity.

Finally, we found a gradient among educational attainment strata in the increase of the percentage of subjects who reported a recent blood sugar check between 2005 and 2009. In effect, subjects with only a primary school education increased their frequency by 18%, compared to 15% in those with a secondary school education and 12% in those with a university-level education (Mantel Haenzel test = 619.2, P<0.001).

## Discussion

In Argentina, reports from the ministry of health expressed concern about the increased prevalence of diabetes between 2005 and 2009, mostly attributing this change to an increase in obesity, consumption of unhealthy foods and low PA [10]. Other studies based on the ANRFS dataset showed that not only the prevalence of diabetes, but also the prevalence of other risk factors such as obesity and low PA changed over this relatively short period of time. The distribution of these risk factors across the socio-economic strata also changed, with increasing signs of inequalities [4]; the distribution exhibited an inverse socioeconomic pattern (i.e., lower risk factor levels in more advantaged groups), which became stronger or only emerged in more urban settings [12].

This study shows that the increase in the reported prevalence of diabetes between 2005 and 2009 in Argentina does not reflect an increase in its actual prevalence but an improvement in the access to preventive care, and better preventive care means also more opportunities to be diagnosed. If this holds true, then the increase in the self-reported prevalence of diabetes in the whole population would be an artifact of more people having blood sugar checks, which in turn could be a surrogate of better access for socioeconomically disadvantaged groups. In fact, the north-west region of the country, which includes the poorest provinces (i.e., Santiago del Estero, Salta and Jujuy) showed a higher increase in the prevalence of self-reported diabetes: 6.5% in 2005 (95% CI, 5.8%;7.2%) and 10.5% in 2009 (95% CI, 9.5%;11.5%), which represents a surprising 60% increase in these provinces. In this regard, subjects with low levels of education exhibited a 50% increase in their frequency of recent blood sugar checks as compared to high-educated subjects (18% vs. 12%) between 2005 and 2009.

On the other hand, self-reported diabetes among people who have recently checked their blood sugar may be considered a better proxy of diabetes when blood samples are obtained, than self-report across the whole population. In this respect, the prevalence of self-reported diabetes did not increase in the subset of people who have recently checked their blood sugar.

Interestingly, as opposed to the increase in the prevalence of self-reported diabetes, the prevalence of self-reported hypertension did not change between the two surveys: 34.5% in 2005 vs. 34.8% in 2009 nor did the percentage of subjects with a blood pressure check in the last 2 years since the survey (a statistically non-significant increase of 3.4%). Therefore, the prevalence of self-reported hypertension among those who had a recent blood pressure check showed no changes either between 2005 and 2009 (36.6% and 35.9%, respectively).

Moreover, even though changes in two major determinants of the prevalence and incidence of diabetes were observed between 2005 and 2009 (e.g., increases in the prevalence of self-reported obesity and low PA), four years is a short time to observe such a large change in the prevalence of a chronic condition such as diabetes. In chronic disease epidemiology, the induction period is the interval between the beginning of the exposure and the first biological presence of the disease while the latent period is the time between the disease's first presence and its recognition [13]. Because chronic conditions require both long-term exposures for induction and long latency periods to diagnoses, it is highly unlikely that the reported change in diabetes prevalence could be due to more obese or more sedentary subjects in 2009 compared to 2005.

Diabetes is often defined exclusively by using a self-reported diagnosis in cross-sectional or prospective studies [14]–[16]. A systematic review found 11 articles describing studies of the validity of self-reported diabetes in survey data compared with reviews of medical charts [17]. Sensitivity ranged from 70% to 99% and specificity ranged from 92% to 99%. ARIC investigators also showed that both prevalence and incidence of self-reported diabetes were 84%–97% specific and 55%–80% sensitive when multiple reference definitions were used [18]. Other studies found even lower sensitivity for self-reported diabetes (66%) [19]. Baseline results of a still unpublished large population-based cohort study in two cities in Argentina conducted by our group show a sensitivity of 64% and a specificity of 99% for self-reported diabetes when compared with direct measures. It is likely that self-reported diagnoses in developing countries are grossly underestimating the actual prevalence of diabetes, as compared to developed countries because of issues related to both health access and health literacy, as was mentioned above. Then, if health access were to improve, awareness should also improve, making self-reported diagnosis flawed to report prevalence. On the other hand, self-reported diabetes in both waves of the ANRFS was measured with a standardized questionnaire developed by WHO [8]. Since most demographic health surveys are aimed at monitoring trends, what is relevant for decision makers is the change rather than the actual estimates, unless, as might be the case here, there are underlying changes that make this trend uninterpretable. Last but not least, neither the frequency of self-reported hypertension nor the frequency of recent blood pressure check went up from 2005 to 2009. In a previous study using data from the ANRFS, we showed that even though access to recent blood pressure measurement in a survey population was related to SES, the strength of the association did not significantly change between 2005 and 2009 [20]. In this regard, it is presumable that access to blood pressure checks in populations with low levels of education preceded access to venipunctures that usually require more complex ancillary services, so no changes were observed in self-reported hypertension in comparison to self-reported diabetes between 2005 and 2009.

Our study has some limitations. First, due to the cross-sectional nature of the surveys, all information on the predictor variables were collected simultaneously with data regarding the outcome: self reported diagnoses of diabetes. This lack of a temporal collection limits the ability of the models to infer causality. Second, we used only education and health insurance status as proxies of SES. Although income status was not included because data in the ANRFS is aggregated at a household and not individual level as the other variables, it has shown to be under-reported among higher income groups in Argentina, which could result in narrower SES gradients [21]–[22]. Third, the models are limited to analysis of individual characteristics. Further analytic approaches could include multilevel techniques to explore how the social gradients in access to preventive services, which confounded the trend observed in self-reported diabetes, could be influenced by contextual effects and ecologic variables at an aggregated level [23]. Lastly, it is important to recognize that the determinants of preventive services, as of any other health service, must be analyzed taking into account the particular health system context in each country. In this regard, the extent of health coverage, the degree of financial protection, the content of the benefit package, and the provider payment schemes and incentives, might affect how preventive services are actually delivered.

In conclusion, the finding of an increased prevalence of self-reported diabetes between 2005 and 2009 might indicate an improvement in the access of disadvantaged subjects to preventive services rather than a positive increase in the prevalence of diabetes. Paradoxically, this could be interpreted as a narrowing and not a widening of the inequity gap in regard to diabetes. In this sense, it would be interesting to investigate whether this improvement in health care access is also reflected in a decreased rate of diabetes-related complications, particularly in low SES population.

In conclusion, policy makers should be cautious when reporting changes or trends in risk factors from health demographic surveys based only on self-reported data.
